# Visual function, spectacle independence, and patients' satisfaction after cataract surgery- a study in the Central Region of Ghana

**DOI:** 10.4314/ahs.v21i1.55

**Published:** 2021-03

**Authors:** Samuel Kyei, Bio Kwadwo Amponsah, Kofi Asiedu, Yaw Osei Akoto

**Affiliations:** 1 Department of Optometry and Vision Science, School of Allied Health Sciences, College of Health and Allied Sciences, University of Cape Coast, Ghana; 2 Eye Clinic, Achimota Hospital, Accra, Ghana; 3 Eye Clinic, Cosmopolitan Medical Center. North-Dwuwulu, Accra, Ghana; 4 Eye Clinic, Our Lady of Grace Hospital, Breman Asikuma, Central Region, Ghana

**Keywords:** TNO, contrast sensitivity, spectacle independence, cataract surgery, intraocular lens

## Abstract

**Background:**

Reduced visual function is associated with diminished quality of life as well as decreased physical and mental health. Poor visual function related to cataracts is also a risk factor for falls and traffic accidents, which may lead to hospital admissions and limit independence.

**Objective:**

To evaluate patients' satisfaction, visual functions and spectacle independence among patients in the Central Region of Ghana who had cataract surgery in one eye.

**Methods:**

A hospital-based prospective cohort study was carried out on 146 patients booked for cataract surgery: 16 were lost through follow-ups whilst 130 completed the study. Visual functions including visual acuity, contrast sensitivity, stereopsis and colour vision were assessed before and after a month of cataract surgery. Objective and subjective refractions were performed to determine the post-surgery refractive status of the participants. Participants completed the NEI-VFQ 25 questionnaire and the scores obtained were used as a construct of their satisfaction.

**Results:**

The NEI-VFQ 25 questionnaire scores indicated patients' satisfaction was high with an average quality of life score of 77.46. Patients satisfaction was strongly correlated with contrast sensitivity (r=0.653, p<0.001) but moderately correlated with visual acuity (r=-0.554, p<0.001), stereopsis (r=0.490, p<0.001) and colour vision (r=0.466, p<0.001). Contrast sensitivity was a better predictor of patients' satisfaction than visual acuity and stereopsis. Spectacle independence at distance was achieved in only 44.6% of the participants and 5.4% at near. There was a significant (p>0.001) association between spectacle independence and the two types of cataract surgery performed which included Small Incision Cataract Surgery (SICS) and Extracapsular Cataract Extraction (ECCE). Among those who were spectacle independent, 53.4% of them were low vision patients.

**Conclusion:**

Satisfaction of patients after cataract surgery was high but was greatly influenced by visual functions with contrast sensitivity being a better predictor of satisfaction than visual acuity and stereopsis. Spectacle independence after cataract surgery was low at distance and extremely low at near. The type of cataract surgery performed influenced thespectacle independence.

## Introduction

Cataracts lead to deterioration of vision, which may restrict activities of daily living. Decreased visual function is associated with diminished quality of life,^1^ as well as a decrease in physical and mental health.^2^ Poor visual function related to cataracts, is a risk factor for falls and traffic accidents, which may lead to hospital admissions and limit independence.^3^,^4^ In spite of this, there is reportedly low uptake of cataract surgery (48.9%) which does not meet the target of vision 2020.^5^ It is expected that after cataract surgery optimal vision will be restored, but some studies have shown low visual outcome after cataract surgery in developing countries.^6^,^7^ Several studies have shown that cataract could diminish visual acuity, contrast sensitivity, stereopsis, and colour vision.^8^

Freedom from spectacle wear has been highlighted as an outcome variable after cataract surgery, not only for the overall patient's satisfaction and quality of life 9 but also for spectacle cost especially to aged people with cataracts. Although after cataract surgery, most patients wish to achieve spectacle independence., unless one chooses presbyopia-correcting IOLs, it's more likely one will need reading glasses after cataract surgery to see near objects clearly. Even people who choose these premium IOLs often find reading glasses helpful for certain near tasks^9^ and in seeing very small print. In the event that there is a refractive error present after surgery, patients may want to wear spectacles especially progressive lenses full-time after surgery to attain the best possible vision at all distances^9^.

Patient satisfaction is a valuable performance indicator for measuring the quality of care delivered by surgeons providing cataract surgery. NEI-VFQ 25 is an international survey that measures the dimensions of self-reported vision targeted health status of patients with chronic ocular conditions such as cataract, glaucoma, macular degeneration, etc. Its reliability and validity have been assessed to be adequate across different geographical locations.^10^,^11^ It comprises 25 questions that can be grouped into 12 main domains: general health, general vision, ocular pain, near activities, distance activities, social functioning, mental health, role difficulty, dependency, driving, colour vision, and peripheral vision. Scores are obtained from the responses given by participants and could be used to determine the quality of life and satisfaction after cataract surgery. The accuracy of the surgery can be achieved consistently through careful attention during the patient selection process, accurate measurement of axial length and corneal power, appropriate selection of an IOL power, etc.^12^ As such, patients' satisfaction highlights the care and diligence with which the surgery is assessed, planned and executed. Failure to achieve this satisfaction after surgery would reflect the patterns of patient selection or treatment that should be assessed for opportunities for improvement.^13^

Visual function and spectacle independence has been highlighted to influence patients' satisfaction.^9^,^14^ Studies have reported that improvement in visual function is associated with high quality of life and satisfaction after cataract surgery.^15^ However, some studies have shown that visual acuity has a limited influence on satisfaction. 16 One study also concluded that stereopsis has no effect on visual task after surgery.^17^ Cochener et. al,^9^ indicated that spectacle independence was strongly associated with patients' satisfaction. However, another study conducted by Calladine and others 18, 19 found higher spectacle independence in a group that received multifocal IOL as compared to a monofocal IOL group even though they had a similar level of satisfaction.

Due to the inconsistencies in results of these studies conducted at different geographical locations, there is the need to evaluate patients' satisfaction and its association with visual function and spectacle independence in Ghana. Thus, there is no evidence in Ghana and African at large as to whether patients' satisfaction is actually based on their objective visual outcome, spectacle independence or the patients' expectations. This would provide a unique jurisdictional situation for comparison. This study therefore, sought to determine and evaluate patients' visual functions, spectacle independence and satisfaction after cataract surgery.

## Materials and methods

A hospital-based prospective cohort study was utilized. This design is ideal for this study because it was essential to get the baseline data of the visual functions of the participants before the surgery and follow them up to repeat the visual functions after the surgery in order to appreciate the improvement in vision. The study population comprised of cataract patients visiting three referral centers in the Central Region of Ghana within the period of the study. The names of these facilities withheld for the purposes of anonymity. Purposive sampling method was used to sample the study participants. The study included patients who have been booked for cataract surgery within the period of the study except those with history of co-morbid and ocular disorders such as diabetes mellitus, glaucoma, retinal lesions and macular degeneration.

### Sample Size

Sample size calculation was done using the sample size formula for finite (small) population.^20^

n=NZ^2^P(1-P)/ d^2^(N-1) + Z^2^P(1-P).

Where;

n' = sample size with finite population correction,

N = size of the target population = 200 (estimated number of cataract surgery registry in the three facilities chosen)

Z = statistic for 95% level of confidence equal to 1.96

P = estimated proportion of cataract uptake in Ghana = 48.9% 5

d = margin of error = 5%

Sample size = 200x1.96^2^x0.489(1-0.489)/0.05^2^(200-1) + 1.962x.489(1-0.489) = 131.77

### Data collection and Procedures

**Data was collected in three parts:**

1.A. Visual function tests were performed on study participants before surgery and in one-month post-surgery, and the results were recorded. These tests included:

i. Visual acuity testing with a Snellen chart at 6m and converted to logMAR values for statistical analysis. Visual acuity such as counting fingers (CF), hand motion (HM), perception of light (PL) and no perception of light (NPL) was replaced with logMAR values 2.10, 2.40, 2.70 and 3.00 respectively.

ii. Contrast sensitivity was measured with a Pelli-Robson chart at a test distance of 3m. Participants were instructed to read the letters starting from the highest contrast until they were unable to read two or three letters on a line. The participant was assigned a score corresponding to the last group of letters in which two or three letters were correctly read.

iii. Stereopsis was tested with a TNO booklet at 40 cm. While wearing a red-green anaglyph spectacle, the participant was introduced to a plate I in which two butterflies were present in a random-dot stereogram; one was only visible when both eyes were used. Plates II, III, IV contained discs of different sizes, geometric shapes, and discs of different colours respectively which the participants were asked to identify. These plates represented the screening and familiarization phase of the test which was assigned a threshold of <1980 arcsec. After this screening phase, the participants were introduced to plate V and VI which contained the levels of disparity: 480, 240,120 and 60 arcsec. The final threshold score was derived from the last level of disparity which was answered correctly.

iv. Color vision was assessed with pseudo-isochromatic Ishihara 14 plates under optimum daylight and temperature conditions. The booklet was placed at a distance of 75cm and participants instructed to read the number on the plate within 2 seconds after which it was turned to the next plate. The number of correct responses was recorded out of the 14 plates. Participants who had 10 or more correct responses were designated as having a normal red-green colour vision and those who had less than 10 responses correct were designated as red-green colour deficient.

B. Improvement in visual functions was calculated as visual function measured after surgery-visual function measured before surgery.ie. logMAR VA after surgery - logMAR VA before surgery.

Visual acuity, contrast sensitivity, and colour vision were measured monocularly (only in the surgical eye), except stereopsis which is strictly a binocular test.

2. The NEI-VFQ-25 questionnaire was self-administered to study participants to complete after surgery and was scored by the researcher according to the scoring manual. The quality of life scores obtained was used as a construct of their satisfaction score. The satisfactory score was used to scale the participants at 5 levels; very satisfied (80-100), satisfied (<80-60), neither satisfied nor dissatisfied (<60-50), dissatisfied (<50-30) and very dissatisfied (<30)

3. Distance and near refraction were performed on each participant a month after the cataract surgery. Participants who did not require to use spectacles to perform their daily activities at far or near distances were termed “spectacle independent at distance” and “spectacle independent at near” respectively. Refraction was done objectively with a retinoscope and subjectively with a trial lens set.

### Ethical consideration

This study was conducted in accordance with the declaration of Helsinki and was approved by the Institutional Review Board (IRB) of the University of Cape Coast (Ethical Clearance ID: UCCIRB/CHAS/2017/48). All ethical principles regarding the conduct of research with human subjects were taken into consideration. When the research received ethical clearance, a formal introductory letter was sent to the Administrators or the Heads of the three facilities chosen. Permission was granted by the Heads of the respective facilities to be conducted in their facilities. All patients who met the inclusion criteria and willing to voluntarily participate signed an informed consent form before they were recruited into the study.

### Statistical analysis

The data were analyzed with the Statistical Package for Social Sciences version 22 for Windows (IBM Inc., USA). Data were entered into the SPSS program and descriptive statistics were used to determine the visual functions of the participants before and after surgery, estimate the proportion with spectacle independence and the level of satisfaction after the surgery. The Chisquare test was used to find associations between variables. A pired sample T-test was performed to determine if there was a significant improvement in visual functions. Pearson's correlation was used to determine whether there was a correlation between patients' satisfaction scores and visual functions. Multiple regression was used to ascertain whether improvement in visual function could predict patients' satisfaction. Statistical significance was considered at p ≤ 0.05.

## Results

### Demographic and Distributive Characteristics

A total of 146 participants were initially recruited from the three referral facilities but 16 of them were lost to follow up. Thus, 52 from facility A, 43 from B and 35 from C took part in the study. There were 47(48.3%) males and 73(56.2%) females. The age range was 15 to 85 years with a mean age of 63.62 13.47 years. Majority of the participants (90%) were above age 50.

There were two types of cataract surgery performed at the three facilities during the period of the study. These were the Small Incision Cataract Surgery (SICS) with Posterior Chamber Intraocular Lens (PCIOL) and the Extracapsular Cataract Extraction (ECCE) with Posterior Chamber Intraocular Lens (PCIOL). Among the participants, 95(73.1%) had SICS+ PCIOL and 35(26.9%) had ECCE+PCIOL. All participants underwent unilateral cataract surgery and were implanted with the conventional monofocal IOLs only during the surgery. More than half, 69(53.1%) of the participants' operated eye was the right eye.

### Visual Outcomes After Cataract Surgery

Visual outcomes were generally low with reference to WHO standards.^6^,^19^ Sixty-eight, 68(52.3%) of the participants had a good vision (VA ≥ 6/18), 54(34.6%) had a borderline vision (VA 6/24-6/60) and 17(13.1%) had a poor vision (VA<6/60). Moreover, ECCE recorded a better outcome than SICS. Among participants who had ECCE about 63% had a good vision whilst only 48.5% of those who had SICS achieved a good vision.

### Pre-and Post-Surgical Refractive Status

Wilcoxon Signed-Rank Test suggested that there was no statistically significant change in mean spherical refractive error pre-and post-surgery but a significant change in mean astigmatism of magnitude -0.350.90 (p=0.001). Pre-and post-surgical change in mean LogMAR visual acuity was found to be -1.470.68 (p=0.001).

After surgery, SICS recorded 26.3% myopia, 8% hyperopia and 33.7% astigmatism. On the other hand, 14.3% myopia, 2% hyperopia, and 71.4% astigmatism were recorded for ECCE.

### Pre- and post-surgical visual functions

#### Visual acuity before and after cataract surgery

The pre-operative visual acuities of the participants' eye to be operated was very poor with 118 (90.7%) of them having a VA of worse than 6/60 and 12(9.3%) having a VA of 6/60 or better. Out of the 118 participants who were visually impaired (VA less than 6/60) before surgery, 62(52.5%) of them had improved visual acuity of 6/18 or better whilst 39(33%) of them had an improvement in visual acuity of 6/60-<6/18 after surgery. Paired sample T-test suggested that there is a statistically significant difference between the means of the visual acuity measured before and after the surgery which justified the improvement that was observed. (t =19.802, p<0.001).

### Contrast Sensitivity before and after Cataract Surgery

The pre-operative contrast sensitivity of the study participants ranged from 0.00 to 1.35. The mean Contrast Sensitivity was 0.11. Majority of the participants, 98(75.4%) had no contrast sensitivity during the preoperative assessment. One month after the surgery, the contrast sensitivity ranged from 0.00 to 1.65 with an average improvement from 0.11 to 0.88. However, all the contrast sensitivity values obtained were below the expected normative values for the Pelli-Robson chart. Majority of the participants, 95(74.6%) had a contrast sensitivity value of 0.75 to 1.65. A paired sample T-test suggested that there was a statistically significant difference between contrast sensitivity before and after the surgery (t =-11.988, p<0.001).

### Colour Vision before and after cataract surgery

Before the cataract surgery, 98 (75.4%) of the participants had a red-green colour defect whilst the remaining 32(24.6%) had a anormal red-green colour vision (participants who identified ≥10 plates out of 14) in the eye to be operated. Out of the 98 who were colour defective before the surgery, 60 (61.2%) had normal redgreen colour vision after the surgery. A McNemar test showed that there was a statistically significant difference between participants with red-green colour deficiency and those with a normal red-green colour vision before and after cataract surgery (p<0.001).

### Stereopsis before and after Cataract Surgery

Before the surgery was performed, the majority of the participants, 69(53.1%) had subtle stereopsis of <1980 arcsecond whilst 50(38.5%) had no stereopsis at all. Only 2(1.5%) participants had normal stereopsis (120-60 arcsec). One month after the surgery, 26% of participants with stereo acuity <1980 arcsec had improved to a better stereo acuity of 120-60 arcsec. However, there were still many of them 52(40%) with poor stereopsis of <1980 arcsec. A pired sample T-test showed there was statistically significant in stereo visual acuity before and after the surgery (t =-11.99, p<0.001).

### Patients' Satisfaction

Most of the participants were satisfied with the outcome of the surgery. Their satisfaction level ranged from a quality of life score of 11.96 to 97.73 with an average of 77.46±17.89. On a 5-point satisfactory scale, 80 (61.5%) of the participants were very satisfied, 30 (23.1%) were satisfied, 6 (4.6%) were neither satisfied nor dissatisfied 10 (7.7%) were dissatisfied and 4 (3.1%) were very dissatisfied. Results from the 12 main domains (table 3) of the NEI-VFQ-25 suggested that participants rated their ability to perform visual task that involves colour (97.60±12.47) and peripheral vision (89.40 22.95) higher than other vision related activities. They appreciated their ability to perform near activities (74.63±27.07) than distance (67.59±33.26). Participants scored their ability to engage in activities involving social functioning (77.53±36.18), mental health (77.28±29.73) and role difficulty (73.90± 28.17) more than their dependency (69.23±33.49) on others to perform visual activities. In addition, it was revealed that participants rated ocular pain (78.97±24.45) and their general vision status (75.85±18.25) as a more important measure of their quality of life than their general health status (63.27±18.73). However, almost all the participants did not engage in driving.

**Table 3:** NEI-VFQ-25 scores for the each of the 12 domains

Domains	Average score
General health	63.2718.73
General Vision	75.85
Ocular Pain	78.9724.97
Near Activities	74.6327.07
Distance Activities	67.5933.26
Social Functioning	77.5336.18
Mental health	77.2829.73
Role Difficulty	73.9028.17
Dependency	69.2333.49
Driving	*
Colour Vision	97.6012.47
Peripheral Vision	89.4022.95

*missing value

### Spectacle Independence

Some 58 (44.6%) of the participants were spectacle independent at distance while 7(5.4%) were spectacle independent at near after the cataract surgery.

### Associations between patients' satisfaction and visual functions before Cataract surgery

Chi-square test indicated no statistically significant association between patients' satisfaction scale and the visual acuity (χ^2^_(48)_ =32.789, p=0.954), contrast sensitivity (χ^2^_(36)_ =16.666, p=0.998), colour vision (χ^2^_(4)_ =6.299, p=0.178) stereopsis (χ^2^_(16)_ =13.018, p=0.671) of study participants before the cataract surgery was performed.

### Associations between patients' satisfaction and visual functions after cataract surgery

There was a statistically significant association between patients' satisfaction and the visual acuity (χ^2^_(48)_ =134.023, p < 0.001), contrast sensitivity (χ^2^_(44)_ =123.985, p<0.001), and stereopsis (χ^2^_(20)_ = 51.528, p<0.001) and colour vision. (χ^2^_(4)_ =19.541, p= 0.001) of study participants after the cataract surgery.

### Correlation between patients' satisfaction and visual functions

A bivariate Pearson's correlation analysis was performed to determine whether patients' satisfaction was correlated with their visual functions. It showed that patients' satisfaction had a strong positive correlation with contrast sensitivity (r=0.653, p<0.001) followed by a moderate positive correlation with logMAR visual acuity (r=-0.554, p<0.001) stereopsis (r=0.490, p<0.001) and colour vision (r=0.466, p<0.001).

### Patients' satisfaction and visual functions

Multiple regression was performed to ascertain whether visual functions could predict patients' satisfaction. Visual acuity, contrast sensitivity, stereopsis and colour vision after cataract surgery could statistically predict patients' satisfaction (F(4) = 15.918, p< 0.001). The regression model was a good fit for the data and could account for 48.1% of the variation that was observed in patients' satisfaction.

The coefficient table of regression showed that contrast sensitivity, logMAR visual acuity, and stereopsis measured after cataract surgery could significantly contribute to the prediction of patient satisfaction except for colour vision. Standardized coefficient values suggested that contrast sensitivity is a better predictor of patients' satisfaction over visual acuity followed by stereopsis.

Association between patients' satisfaction and spectacle independence Chi-square analysis suggested that there was no statistically significant association between patients' satisfaction and spectacle independence at both distance and near.

### Association between spectacle independence and the type of surgery

A chi-square analysis was performed to determine whether spectacle independence was associated with the type of cataract surgery performed. It was found that there was a statistically significant association (χ^2^_(1)_ =11.124, p=0.001).

Small Incision Cataract Surgery (SICS) achieved a better spectacle independence at distance (31.6%) than Extracapsular Cataract Extraction (ECCE) (11.4%).

## Discussion

**Figure 1:**
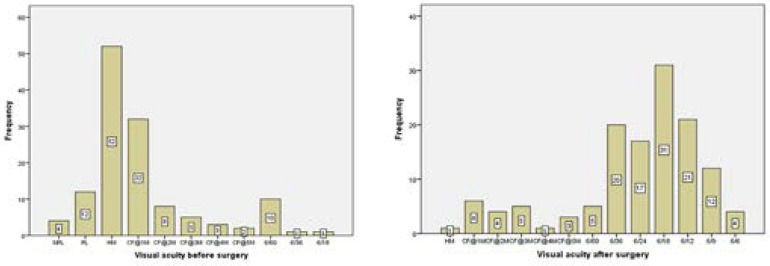
A bar chart showing the frequency distribution of visual acuity (a) before cataract surgery, (b) after cataract surgery.

**Figure 2:**
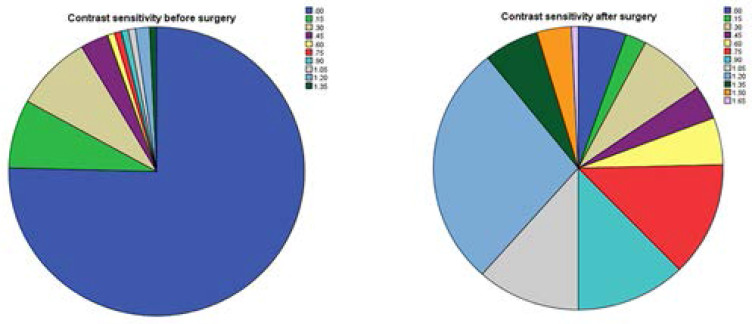
A bar chart showing the frequency distribution of contrast sensitivity (a) before cataract surgery, (b) after cataract surgery.

**Figure 3:**
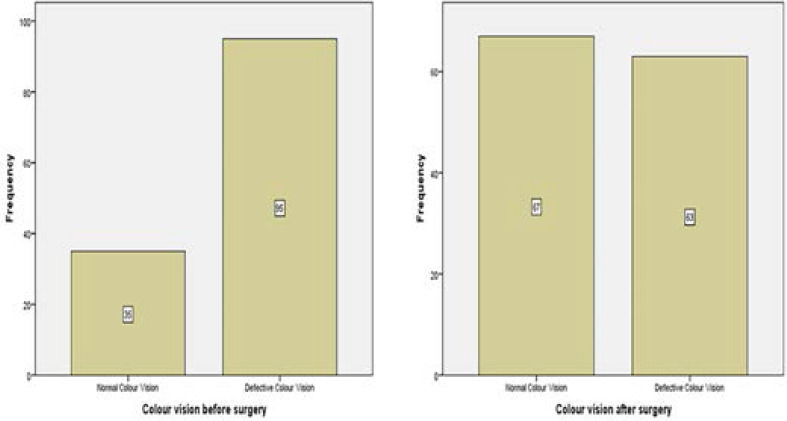


**Figure 4:**
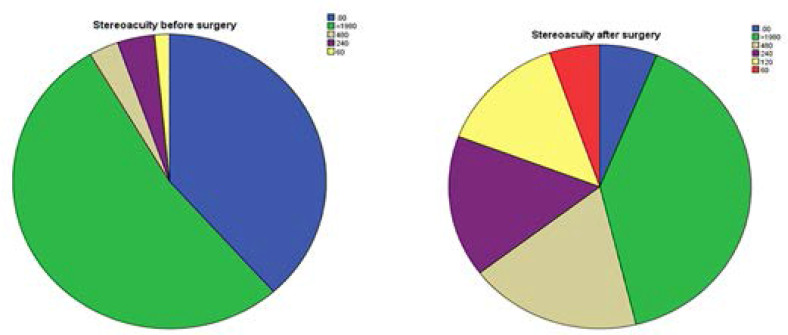
A bar chart showing the frequency distribution of stereo acuity (a) before cataract surgery, (b) after cataract surgery.

**Figure 5:**
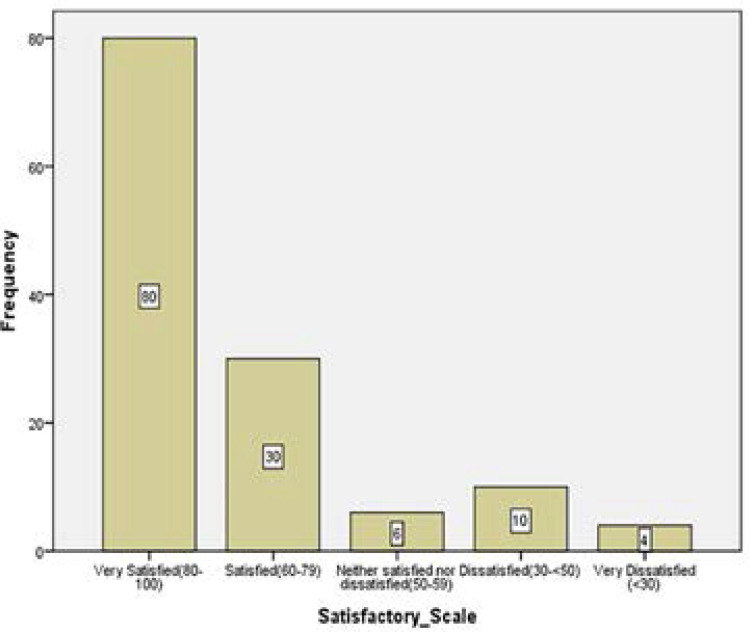
A bar chart showing the frequency of patients' satisfaction on a 5-point scale.

Cataract is the major cause of blindness in most countries in the world.^21^,^22^ It is expected that after cataract surgery the patient may gain sufficient vision which was otherwise obstructed by the cataract to be able to undertake visual activities. The pre-operative visual acuity, contrast sensitivity, colour vision and stereopsis of the study participants were very poor in the eye to be operated (Figure 1,2,3 and 4). One month after the cataract surgery, there was a significant improvement in the visual functions measured (Figures 1, 2, 3 and 4) even though the visual outcome was poor with reference to the WHO standards (>85% and <5% for good and poor outcomes respectively).^6^,^21^ This study revealed that most of the patients that underwent cataract surgery during the period of the study were satisfied with their visual improvement after surgery with an average quality of life score of 77.46 (Figure 5). A statistically significant association was found with all the four visual functions measured. This finding was consistent with some studies which reported that improvement in visual acuity is associated with high quality of life and satisfaction after cataract surgery.^13^,^23^

The improvement in visual functions observed could be used by the surgeon to propose a reflection of the patients' satisfaction with the surgery from their point of view.

In this study, it was ascertained that contrast sensitivity had the strongest correlation with the patients' satisfaction followed by visual acuity, stereopsis, and colour vision (Table 1). Multiple regression showed that the visual functions measured after the surgery can significantly predict the satisfaction of the patient. The regression coefficient table suggested that the most likely predictor of patients' satisfaction is contrast sensitivity more so than the visual acuity and stereopsis (Table 2). Meanwhile, the patient's visual expectation that actually determines his satisfaction may be on the contrary because the individual domain scores of the NEI-VFQ-25 shows that participants appreciate their ability to perform a visual task that involves colour differentiation than any other visual related task (table 3). For this reason, patients' satisfaction should usually be measured subjectively to underscore the core expectations of the patients that need to be addressed by the surgeon.

**Table 1:** Pearson correlation between patients' satisfaction and visual functions

	Pearson correlation	Sig(2-tailed)
LogMAR VA after surgery	-0.554**	0.000
Stereoacuity after surgery	0.490**	0.000
Contrast sensitivity after surgery	0.653**	0.000
Colour vision after Surgery (number of plates identified)	0.466**	0.000

** Correlation is significant at the 0.01 level (2-tailed)

**Table 2:** Multiple regression between patients' satisfaction and visual functions

Model	Unstandardized Coefficients		Standardize Coefficients		Sig.
	B	Std. Error	Beta	t	
(Constant)	62.408	5.070		12.309	0.000
LogMAR VA after surgery	-5.737	2.049	-0.231	-2.799	0.006
Colour vision after Surgery (number of plates identified)	-0.396	0.474	-0.081	-0.834	0.406
Contrast sensitivity after surgery	21.413	5.045	0.478	4.244	0.000
Stereo acuity after surgery	2.101	1.043	0.160	2.015	0.046

Dependent Variable: Satisfactory Score

Several studies have reported that improvement in visual acuity is associated with high quality of life and satisfaction after cataract surgery^13^, indicating the importance of visual acuity in everyday life^24^ and after cataract surgery^25^. However, some studies have shown that visual acuity has a limited influence on satisfaction.^14^ Contrary to this study, another research concluded that stereopsis has no effect on visual task after surgery.^15^

In addition, there was the need to study how spectacle independence influence the satisfaction of patients in the Ghanaian setting because a study by Illechie et. al,^6^ highlighted that most patients (90.4%) in Ghana were not refracted after cataract surgery which could account for poor visual outcomes after cataract surgery and probably affect their satisfaction.^6^ This study found that spectacle independence was not a valuable outcome that could influence patients' satisfaction after surgery. There was low spectacle independence at distance and extremely low spectacle independence at near which may be due to the type of IOL used.^18^ Conventional monofocal IOLs which could not cater for presbyopia were implanted for all the study participants. However, out of the 58 patients who were spectacle independent at distance, 31(53.4%) were still low vision (VA of 6/18 or less in the best-corrected eye). There was no significant association between patients' satisfaction and spectacle independence which indicates that patients in Ghana do not consider the need to wear or not to wear spectacles after cataract surgery as an important measure of their satisfaction. On the contrary, Cochener et. al,^9^ found that spectacle independence was strongly associated with patients' satisfaction. However, another study by Calladine et. al,^18^ found higher spectacle independence in a group that received multifocal IOL as compared to a monofocal IOL group even though they had a similar level of satisfaction. Furthermore, this study found a significant association between spectacle independence and the type of cataract surgery with SICS achieving better spectacle independence than ECCE. It has been underscored in the literature that modern techniques of cataract surgery induce less refractive error (particularly astigmatism) than older methods such as the SICS and ECCE which could explain this finding.^25^ A study by Ang et. al, 26 indicated that surgically induced astigmatism was more common with ECCE than SICS. For these reasons many patients who undergo ECCE will require to use spectacles after surgery. However, other reasons such as wrong calculations of IOL power and surgical complications that can affect the refractive status of the patient cannot be overlooked.^27^ The strength of this study over other similar prospective studies is that the research was done at three referral facilities to ascertain whether the different types of cataract surgery commonly practiced in Ghana could have an influence on patients' satisfaction or spectacle independence. Also, this study is the only prospective cohort study that has provided data on patients' satisfaction and spectacle independence after cataract surgery in the African population. The major limitation of this study is that it only used patients who had surgery to one eye. It is not clear what the refractive status or visual acuity was for the non-surgical eye. For a QoL survey, one will expect some differences if the survey was conducted amongst participants who had both eyes treated.

## Conclusion

Patient satisfaction is high and strongly related to visual functions after cataract surgery. Therefore, in addition to visual acuity measurements which have always been the routine visual assessment in most clinics in Ghana other visual functions especially contrast sensitivity measurements should be given due consideration as part of the routine pre-and post-surgical visual assessment of cataract patients.

Spectacle independence after cataract surgery is low at both far and near and could be influenced by the type of cataract surgery performed. Modern methods of cataract surgery such as phacoemulsification and the use of multifocal and toric IOLs should be encouraged in Ghana in order to increase spectacle independence after cataract surgery. Moreover, the role of the optometrist to provide optical correction cannot be underestimated.

Some patients who undergo cataract surgery remain low vision patients after refractive correction. It is recommended that such patients are provided with visual rehabilitation and low vision services.

## Data Availability

The data used to support the findings of this study are included in the article.
